# Pancreatic secretory trypsin inhibitor reduces multi-organ injury caused by gut ischemia/reperfusion in mice

**DOI:** 10.1371/journal.pone.0227059

**Published:** 2020-01-10

**Authors:** Raymond J. Playford, Tania Marchbank

**Affiliations:** 1 Plymouth University Peninsula Schools of Medicine and Dentistry, Plymouth, United Kingdom; 2 Centre of Immunobiology, Blizard Institute, Barts and The London School of Medicine, Queen Mary, University of London, London, United Kingdom; National Institutes of Health, UNITED STATES

## Abstract

Intestinal ischemia/reperfusion (I/R) injury occurs during transplantation, mesenteric arterial occlusion, trauma and shock, causing systemic inflammation, multiple organ dysfunction and high mortality. Pancreatic secretory trypsin inhibitor (PSTI), a serine protease inhibitor expressed in gut mucosa may function as a mucosal protective/repair peptide. We examined whether PSTI affected mesenteric I/R-induced injury. Hypoxia/normoxia (H/N) caused 50% drop in cell viability of AGS, RIE1 and Caco-2 cells but PSTI (10 μg/ml) given prior- or during-hypoxic period improved survival by 50% (p<0.01). Similarly, Caco-2 monolayers exposed to H/N had 300% increase in transepithelial permeability, PSTI truncated this by 50% (p<0.01). Mice underwent mesenteric I/R by clamping jejunum, causing severe mucosal injury, increased apoptotic markers and 3-fold increases in plasma IL-6, IL1β, TNFα, and tissue lipid peroxidation (MDA) and inflammatory infiltration (MPO) levels. Lungs showed similar significant injury and inflammatory infiltrate markers. Smaller increases in MDA and MPO were seen in kidney & liver. PSTI (20 mg/kg) reduced all injury markers by 50–80% (p<0.01). In vitro and in vivo studies showed PSTI reduced pro-apoptotic Caspase 3, 9 and Baxα levels, normalised Bcl2 and caused additional increases in HIF1α, VEGF and Hsp70 above rises caused by I/R alone (all p<0.01). PSTI also prevented reduction of tight junction molecules ZO1 and Claudin1 (all p<0.01) but did not affect increased ICAM-1 caused by I/R in gut or lung. PSTI may be a useful clinical target to prevent I/R injury.

## Introduction

Gastrointestinal ischemia/reperfusion (I/R) injury is involved in multiple clinical situations, such as neonatal necrotizing enterocolitis, acute mesenteric ischemia, volvulus, trauma, cardiopulmonary disease, hemorrhagic shock, and intestinal transplant rejection [1–4]. In addition to local tissue injury, remote organs are damaged by the uncontrolled inflammatory response resulting from release of inflammatory mediators and activation of leukocytes due to the post-ischemic gut serving as a priming bed for circulating polymorphonuclear cells [5, 6]. There is also interplay between the inflammatory process and periods of localized tissue hypoxia in conditions such as inflammatory bowel disease where transmigrating neutrophils rapidly deplete the local gut microenvironment of oxygen [7].

In severe cases, the combination of localised injury with an uncontrolled systemic inflammatory response causes a breakdown in gut mucosal integrity, increased gut permeability and leakage of luminal bacteria and other contents into the circulation. This further exacerbates the injury process, potentially leading to multiple organ failure (MOF) with a mortality rate of up to 80% [8]. Current therapeutic options are limited, consisting of general supportive measures in combination with antimicrobials. There is therefore a need for novel therapeutic interventions.

Growth factors, whether produced by purification or using recombinant technology, are increasingly being used for a variety of clinical conditions. Examples include recombinant human insulin, erythropoietin, and granulocyte-colony stimulating factor (G-CSF). The use of growth factors for ‘hollow organ’ gastrointestinal conditions is, however, at a more preliminary stage.

Pancreatic secretory trypsin inhibitor (PSTI), also known as serine protease inhibitor Kazal type 1 (SPINK1), is a 56-amino acid peptide that protects the pancreas from autodigestion due to premature activation of pancreatic proteases [9]. PSTI expression also occurs in the normal human breast, in human colostrum, and in the mucus-producing cells of the mucosa of the stomach and large intestine [10,11]. It is markedly upregulated in epithelial and other cells in the small intestine at sites of injury, such as in Crohn’s disease, where it is also expressed in the ulcer associated cell lineage [10, 11]. We have shown that transgenic mice that overexpress PSTI in the proximal small intestine have reduced sensitivity to NSAID-induced damage, although the molecular mechanisms involved were not investigated [12]. We also have shown that pro-migratory protective effects of PSTI are not related to its protease inhibitory activity as a site directed mutagenic variant of PSTI which had its serine protease active site disrupted maintained its promigratory effect and soya bean trypsin inhibitor, which is a related serine protease inhibitor, does not stimulate cell migration [10, 13].

In the current series of studies, we extend our previous work to determine whether PSTI administration influences gut I/R-induced injury and examine likely molecular pathways involved in any effects seen.

## Materials and methods

Chemicals were purchased from Sigma (Poole, Dorset) unless otherwise stated.

### Cell lines

Caco-2 is derived from colorectal adenocarcinoma of 72-year-old male (ATCC) and exhibits tight junctions and desmosomes between adjacent cells and grows as polarized monolayers [14]. AGS is derived from gastric adenocarcinoma of a 54-year-old female [15](ATCC), RIE1 is a spontaneously immortalized rat intestinal epithelial cell line [16] (ATCC).

### Hypoxia (Ischemia)/ Normoxia (Reperfusion) (H/N) protocols for in vitro studies

For all experiments AGS, Caco-2 and RIE1 cells were set up in plates as described in specific methods. Cells were used at 70–80% confluence for cell injury/death assessment and as confluent monolayers for permeability studies. To induce “ischemia “, cells were placed in a hypoxic incubator (2% O_2_) for specified time and to mimic reperfusion, cells were placed back in a normoxic (21% O_2_) incubator for specified time. All treatments were duplicated in a second set of identical plates under normoxic conditions throughout the same test period as controls. To determine optimum conditions for the in vitro studies, pilot studies were performed.

### Cell viability (MTT) assay

Cell viability following H/N was assessed using an MTT assay [17]. The principal of the assay is that viable cells contain NAD(P)H-dependent oxidoreductase enzymes which reduce the MTT reagent to formazan which can be measured spectrophotometrically. At the end of the H/N period, 5 mg/ml MTT in PBS was added to each well of the 96 well plates and incubated at 37°C for 4 hours, the medium was replaced with 150 μl of DMSO and absorbance measured at 490nm. Cell viability was expressed as percentage of the equivalent normoxic control, all results are expressed as mean +/- SEM of six wells.

### Lactate dehydrogenase (LDH) assay

Cell damage was assessed using the activity of LDH released into the cell culture medium. LDH is a cytosolic enzyme present in many different cell types. Plasma membrane damage releases LDH into the cell culture media. At the end of the H/N period, supernatant was collected from all wells and LDH activity determined using a Pierce LDH Cytotoxicity Assay Kit (Thermo Fisher Scientific, Hemel Hempstead, UK) following manufacturer’s instructions. Results are expressed as LDH activity and presented as mean +/- SEM of six wells.

### Pilot studies

To test the effect of hypoxia (mimicking ischemia-oxygen deprivation) with and without glucose depravation, followed by subsequent normoxia (mimicking reperfusion), cells were set up in 96 well (for cell viability studies) & 24 well (for subsequent permeability studies) plates and subjected to hypoxia for 1, 4 or 8 hours with and without glucose and then were then returned to normoxia in normal glucose containing medium for 0, 1, 4, 8, or 12 hours. Cell Viability (MTT assay) and cell damage (LDH assay) were assessed.

For all three cell lines, we found 4 h hypoxia followed by 24h normoxia in the absence of glucose gave optimum conditions for establishing a cell survival rate of approximately 50% (S1A Fig) with a three-fold increase of LDH (S1B Fig). For studies examining changes in transepithelial permeability, a protocol of 1 h hypoxia and 24 h normoxia was used as these conditions resulted in a 500% increase in horse radish peroxidase (HRP) permeability but did not affect cell death, allowing a continuous monolayer to be maintained.

### Main studies

#### Study series 1. Effect of PSTI on cell survival

1A: Dose response study: To examine the effect of PSTI on H/N-induced cell injury in vitro, cells were set up in 96 well plates and pre-incubated in SFM containing glucose, with or without 5, 10 or 15 μg/ml PSTI for 1 hour (i.e. PSTI pre-treatment). These concentrations of PSTI were chosen based on our previous studies examining cell migration [12]. The medium was replaced with SFM without glucose and placed in the hypoxia incubator for 4 hours. At the end of the hypoxia period, medium was replaced with SFM containing glucose for 24 hours at normoxia for the reperfusion period.

1B: Efficacy of PSTI given pre-, during- or post-hypoxic period. We compared the potential benefit of giving PSTI prior to, during or following the hypoxic period. Cells were pre-treated with 10 μg/ml PSTI for the 1 hour before hypoxia (Pre), at the time of the start of hypoxic period (During) or added at the start of the return to normoxia stage (Post). Cell Viability (MTT assay) and cell damage (LDH assay) were assessed.

#### Study series 2. Effect of PSTI on Transepithelial Permeability

Background to methods: We examined the effect of 10 μg/ml PSTI pre-treatment on transepithelial permeability induced by 1 hour of hypoxia followed by 24 hours of normoxia using Caco-2, polarising colonic adenocarcinoma cells utilising two different previously published methods [18]. One determined change in transepithelial electrical resistance (TEER) and the other analysed the passage of horseradish peroxidase (HRP) across the epithelial layer. HRP (type II) is a non-digestible macromolecular protein (MW = 44 kD) and has previously been used as a tracer in evaluation of epithelial permeability. Measurements were taken in triplicate from 4 wells per treatment. Results are expressed as mean +/- SEM.

#### Study series 3. Effect of PSTI on gut and distant organ injury in response to small intestinal I/R

Animal Experiments

All animal experiments were approved by local animal ethics committees (QMUL’s Animal Welfare and Ethical Review Board) and covered by the appropriate project licences under the Home Office Animals Procedures Acts, 1986. Experiments were performed under terminal anethesia using ketamine and xylaxine.

I/R model.

C57BL/6 mice (20–25 g) were randomly assigned to one of three groups (n = 6–9 per group); a) I/R alone, B) I/R + PSTI (20 mg/kg, ip.) treatment 1 hour before the clamping procedure of the I/R protocol, C) Sham group animals, that underwent a laparotomy but no clamping of the SI.

Method of induction of I/R: Mice were anesthetised with ketamine (100 μg/g, ip) and xylazine (10 μg/g, ip) and placed on warming mats. Anaesthesia was maintained by intermittent additional doses based on toe pinch response. To induce ischemia, the midsection of SI along with its associated mesenteric vessels, comprising approximately the region 30–75% of total length (where entire length is defined as 100%, with proximal duodenum starting at 0% and end of SI as 100% distance) was clamped using non-traumatic surgical clamps (UK Quality Instruments, Ramsgate, Kent) for 30 minutes, followed by removal of the clamp and 3 hours of reperfusion. The wound sites were kept covered and mice kept anesthetised and warm throughout the reperfusion period. Visual inspection of the previously clamped region of the gut was performed at the time of clamp removal to check that the segment became re-perfused.

At the end of the study, under terminal anaesthesia mice were killed by cervical dislocation and blood collected by cardiac puncture. For intestinal tissues, the position of the various samples was defined by expressing its harvest site as a percentage total length at 50% and 90% small intestinal distance and can be considered as equivalent to jejunum and ileum respectively. The 50% SI collection sample was, therefore, in the middle of the clamped region that had undergone I/R and the 90% site was outside of the previously clamped region. Kidney, liver and lung tissue were also collected. All tissue harvested was split into two, half being snap frozen at -80°C for further analysis, and the other half immediately fixed in 10% neutral buffered formalin for histopathological assessment.

To examine the changes in plasma PSTI levels over time, in addition to measuring PSTI levels at the end of the I/R protocol, additional animals received PSTI injection without I/R and killed at 30 min, 60 min, 90 min post injection.

Histopathological assessment

Tissue was stained using hemotoxylin-eosin and scored in a blinded manner. Intestinal damage was assessed using the scoring method of Chiu et al [19] on a scale of 0 (normal) to 5 (damaged severely). 0- Normal mucosa and villi; 1- Development of subepithelial Gruenhagen’s space at villus tips, often accompanied by capillary congestion; 2- Extension of subepithelial space with moderate lifting of epithelial layer from lamina propria; 3- Massive lifting down sides of villi, some denuded tips; 4- Denuded villi, with lamina propria and dilated capillaries exposed, increased cellularity of lamina propria may be seen; 5- Digestion and disintegration of the lamina propria, haemorrhage and ulceration.

Lung damage was scored using the method of Koksel et al [20] on a scale of 0 (no damage)– 3 (severe damage). 0 -no damage; 1- mild neutrophil leukocyte infiltration and mild-moderate interstitial congestion; 2- moderate neutrophil leukocyte infiltration, perivascular edema formation and disintegration of the pulmonary structure; 3- dense neutrophil leukocyte infiltration and absolute destruction of pulmonary structure. Results are expressed as mean +/- SEM.

Tissue processing for biochemical analysis.

Samples were homogenised on ice in ice-cold 50mM potassium phosphate buffer (pH 6.0) containing 0.5% hexadecyl trimethyl ammonium bromide (HTAB), freeze thawed three times and briefly sonicated. An aliquot of the lysate was stored at -80°C and the remainder centrifuged at 15,000 rpm for 20 min at 4°C. Supernatants were collected and saved as cleared lysates. Total protein concentration of the lysates and cleared lysates was determined using a standard protein assay.

Myeloperoxidase (MPO) assay.

MPO activity, used as a marker of neutrophilic infiltration, was measured as described previously [21]. Cleared tissue lysate was incubated with O-dianisidine dihydrochloride (Sigma) and 0.0005% hydrogen peroxide and change in absorbance at 460 nm recorded using a spectrophotometer. One unit of MPO activity was defined as that consuming 1 nmol of peroxide per minute at 22°C. Results are expressed as U/μg of total protein, mean +/- SEM from triplicate wells.

Malondialdehyde (MDA) assay

MDA levels were determined as a marker of lipid peroxidation from tissue lysates using the thiobarbituric acid method [22]. Results are expressed as nmol MDA/μg of total protein, mean +/- SEM from triplicate wells.

Blood processing for LPS, cytokines and PSTI levels.

Blood was collected into EDTA treated tubes and centrifuged for 10 min at 3,000 rpm. Plasma was collected and frozen until analyses. LPS, IL6, IL1βTNFαand human PSTI were analysed by commercial Elisa’s (Antibodies-online.com) as per manufacturer’s instructions

#### Study series 4. Mechanisms of action of PSTI in the in vitro and in vivo studies

Having shown that PSTI reduced cell and organ damage in our in vitro and in vivo studies, samples from in vitro cell viability studies and whole animal I/R tissues were further analysed to examine PSTI’s possible modes of action.

Cell apoptosis assays

Active caspase-3 and active caspase-9 were determined using methods described previously [18], using commercial colorimetric assay kits (BF3100 and BF10100, R&D Systems). Concentrations of the anti-apoptotic protein Bcl2 and the pro-apoptotic protein Baxα were determined in the same cell lysates as used for caspase analyses, using Duoset Elisa kits (R&D Systems Europe Ltd).

Measurements of HIF1α, VEGF, Hsp70 and ICAM-1

HIF1α, VEGF, Hsp70 and ICAM1 concentration in the cleared cell lysates was determined using Duoset Elisa kits as per the manufacturer’s instructions (R&D Systems Europe). Hsp70 concentrations were determined using our previously published methods [18] with a Duoset Elisa kit (R&D Systems Europe).

Tight junction proteins

ZO1 and Claudin1 concentrations were determined using previously published methods [18] and standard ELISA kits (tight junction antibody samples pack 90–1200, Invitrogen)

### Statistical analyses

All values are expressed as the mean +/- SEM unless otherwise stated. Data were analysed using a one-way ANOVA. Where a significant effect was seen (p < 0.05), individual comparisons were performed using t-tests based on the group means, residual and degrees of freedom obtained from the ANOVA, a method equivalent to repeated measures analyses.

## Results

### Study series 1. Effect of PSTI on cell survival

**Fig 1:**
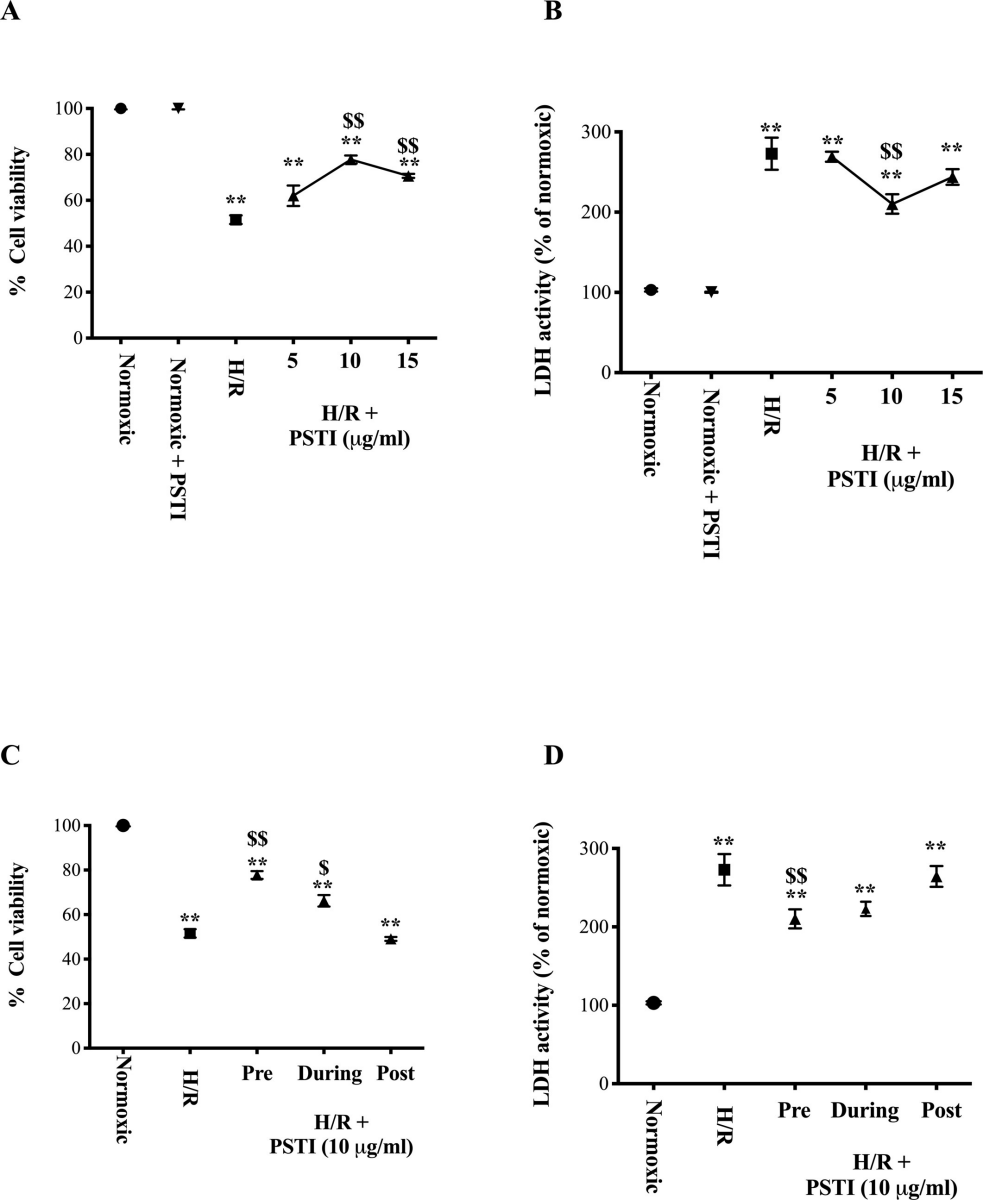
Effect of hypoxia-normoxia (H/N) on cell viability and injury in presence and absence of PSTI. Cell viability using MTT assay (A, C) and cell damage measuring LDH activity (B, D) were determined for cells exposed to 4h hypoxia (ischemia) and then returned to normoxia for 24h (reperfusion). Values are expressed as percentage difference compared to cells incubated in serum free medium alone that were not exposed to H/N. A&B. Cells incubated under normoxic conditions throughout (●), grown in presence of PSTI (10 μg/ml) but not exposed to H/N (▼), subjected to H/N alone (■) and incubated in various concentrations PSTI 1 h prior to undergoing H/N (▲). C& D) The effect of adding PSTI (▲) pre hypoxia, during hypoxia or post hypoxia was compared to cells exposed to H/N alone (■). Results shown are for AGS cells, RIE1 and Caco-2 cells gave similar results. Results expressed as mean +/- SEM. ** signifies p < 0.01 vs normoxic throughout, $ $ signifies p < 0.01 vs H/N alone.

1A: Dose response study: Cells grown under normoxia showed no change in viability or LDH if PSTI was administered. PSTI given prior to H/N caused reduced cell death (MTT and LDH assays) with optimum concentration at 10 μg/ml. At this concentration, cell viability increased from 52 ± 2% to 78 ± 2% (Fig 1A) and the rise in LDH was reduced by 37% ± 2% (Fig 1B).

1B: Efficacy of PSTI given pre-, during- or post-hypoxic period. PSTI (10 μg/ml) administered pre-hypoxia, during hypoxia or post-hypoxia (at the reintroduction of normoxia stage) showed greatest efficacy was seen if PSTI is given pre-hypoxia (Fig 1C). Similar reciprocal changes were seen following changes in LDH (Fig 1D).

### Study series 2. Effect of PSTI on TEER and transepithelial permeability

**Fig 2:**
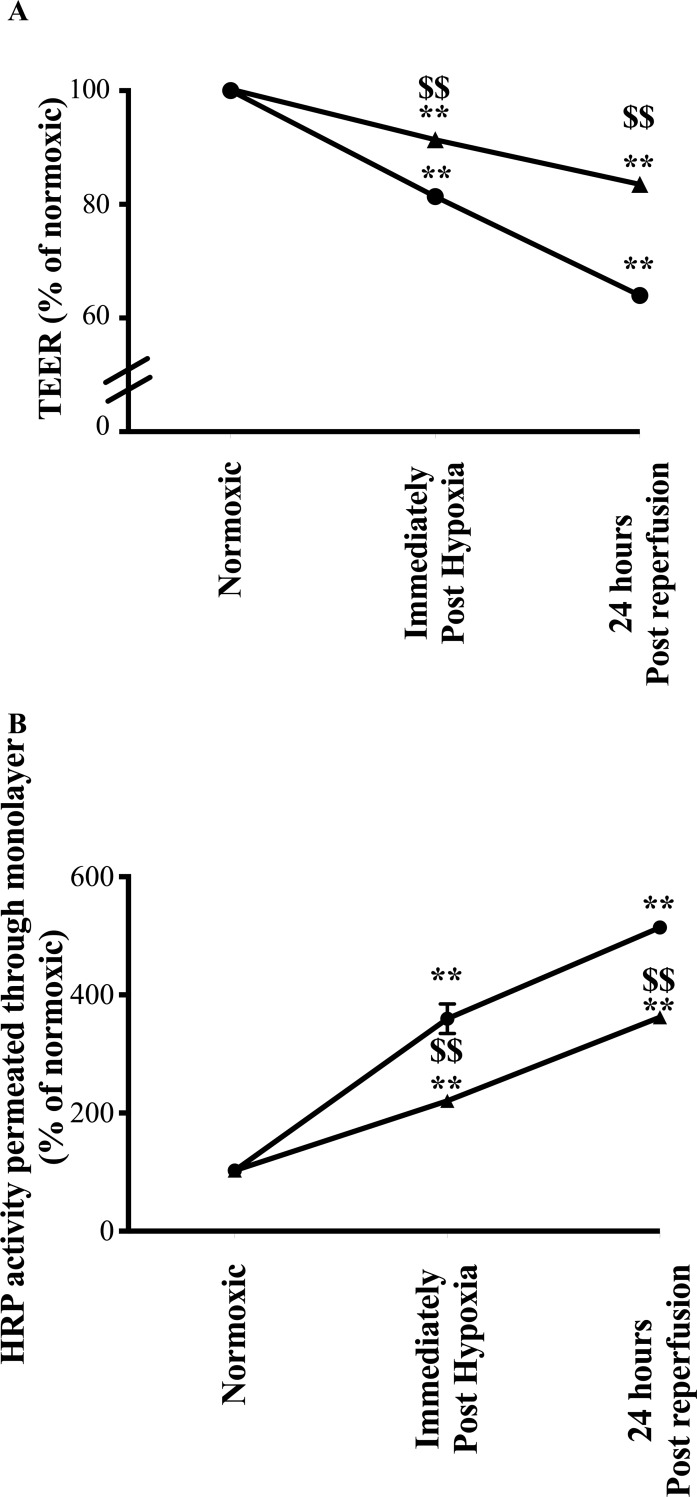
Effect of PSTI on H/N- induced changes in TEER and transepithelial permeability. Polarised Caco-2 monolayers were exposed to H/N and the effect of PSTI on changes in TEER (A) and permeation of HRP through the monolayer (B) determined. Each monolayer had TEER and HRP permeability assessed at baseline (“normoxic”), at the end of 1h incubation in a hypoxic chamber and at the end of a 24h reperfusion period. Monolayers incubated in serum free medium alone (●) had significantly greater increases in permeability compared to those that had PSTI added to the medium prior to H/N (▲). ** signifies p < 0.01 vs baseline normoxia values, $ $ signifies p < 0.01 vs values seen in cells incubated in medium alone.

The presence of PSTI given prior to H/N had no effect on TEER or HRP permeability under normoxic conditions. In monolayers subjected to H/N in the absence of PSTI, TEER fell to 81.3 ± 0.28% at the end of the hypoxia period and fell further to 63.9 ± 0.49% at the end of the H/N period. Pre-treatment with PSTI reduced the fall in TEER caused by H/N by about 50% (Fig 2A).

Similar reciprocal results were seen following permeability of HRP across monolayers. Control cells in serum free medium alone had a 3-fold increase in HRP permeability immediately after the hypoxic period, rising to a 5-fold increase at the end of the H/N period. The presence of PSTI truncated the effect of hypoxia by about 54% at the end of the hypoxic period and by 37% at the final H/N time point (Fig 2B).

### Study series 3. Effect of PSTI on gut and distant organ injury in response to small intestinal I/R

#### Histological damage and MPO and MDA tissue levels

**Fig 3:**
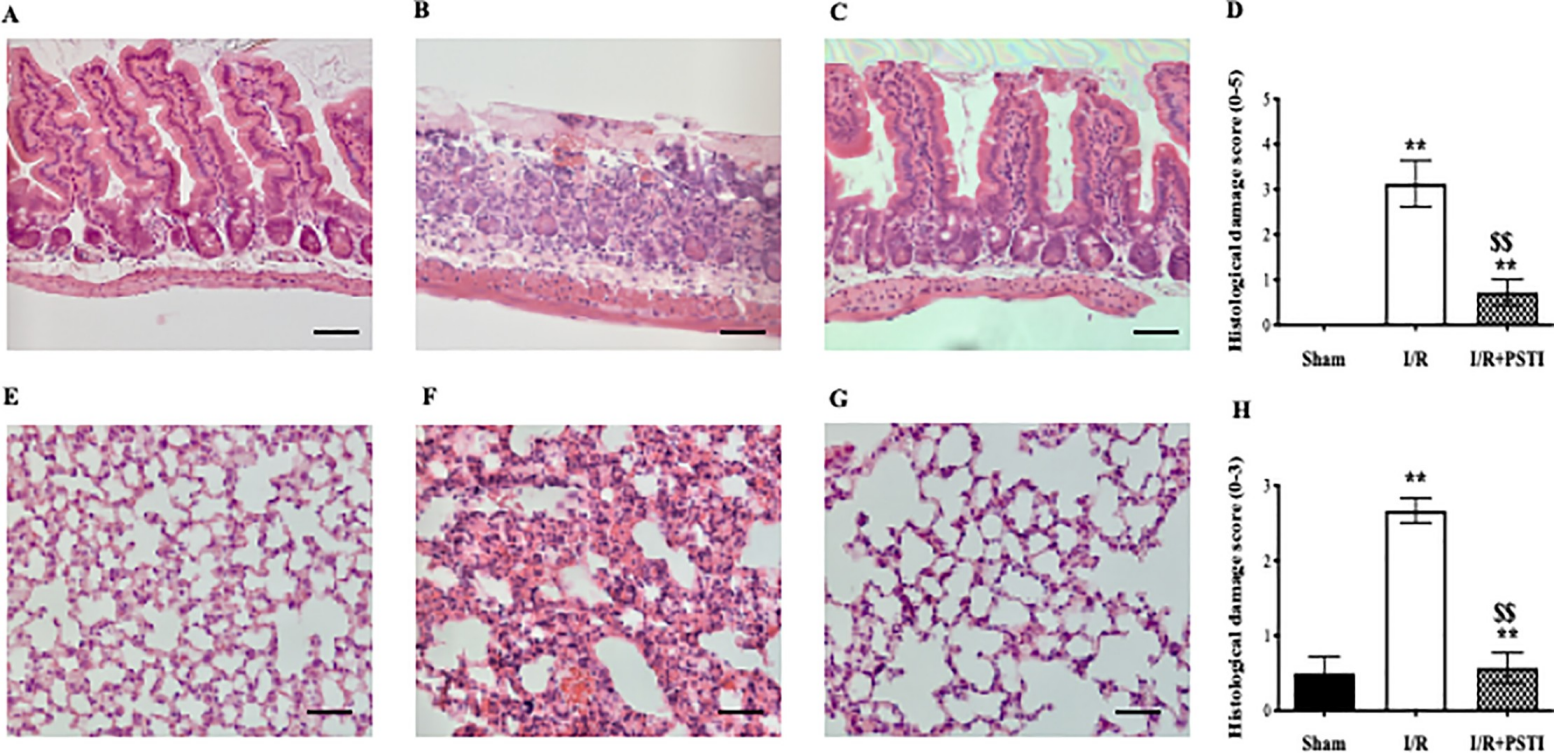
Effect of PSTI pre-treatment on histology and damage score in SI and lung following I/R. Mice underwent a sham (laparotomy only) procedure or subjected to 30 min mesenteric ischemia followed by 3 hours of reperfusion (I/R). Some animals also received PSTI (20 mg/kg, ip) 1 hour before gut clamping (I/R + PSTI). Photomicrographs (original magnification 200X, scale bar = 100 μM) of intestinal tissue from 50% SI region **A)** sham operated control animal, **B)** animal that had undergone I/R protocol, **C)** animal pre-treated with PSTI prior to I/R. Photomicrographs of lung tissue (original magnification 200X, scale bar = 100 μM) from **E)** sham operated control animal, **F)** animal that had undergone I/R protocol, **G)** animal pre-treated with PSTI prior to I/R. Formal histological damage scoring using methods of Chiu[19] for SI damage **(D)** and Koksel20[20] for lung damage **(H)** showed PSTI pre-treatment reduced I/R-induced injury. ** signifies p < 0.01 vs sham operated animals, $ $ signifies p < 0.01 vs I/R alone.

Intestinal tissue from 50% SI region of sham operated control animals showed normal histology (Fig 3A), whereas the same region of animals that had undergone I/R showed extensive denudation and loss of villus structure and inflammatory infiltration (Fig 3B). In contrast, animals that had received PSTI prior to I/R showed much less extensive damage (Fig 3C). Tissue from the 90% SI length (not directly subjected to I/R) appeared histologically normal in all groups.

Lung tissue from sham operated control animals had normal histology (Fig 3E), whereas the I/R group showed marked pulmonary congestion and diffuse interstitial inflammatory cell infiltrate and areas of tissue destruction (Fig 3F). PSTI pre-administration markedly truncated these effect (Fig 3G).

Formal histological damage scoring using methods of Chiu[19] for SI damage (Fig 3D) and Koksel[20] for lung damage (Fig 3H) confirmed histological appearance with PSTI pre-treatment reducing intestinal injury (50% site) and lung injury by approx. 80% (both p<0.01 vs I/R alone).

Similar results were seen when MPO & MDA levels were assessed (Table 1). In both 50% SI and lung, MPO & MDA levels were increased in response to I/R and this increase was truncated in animals pre-treated with PSTI. Assessment of tissue from the 90% SI site (not directly subjected to I/R) showed no changes in MPO or MDA following I/R with or without PSTI pre-treatment.

**Table 1:** Effect of gut ischemia-reperfusion (I/R) +/- pre-administration of PSTI on injury and protective pathways in 50% SI, lung, renal and liver tissue. Data presented as mean +/- SEM. * and ** signifies p<0.05 and p <0.01 vs Sham operated animals (laparotomy only), $ $ signifies p<0.05 and <0.01 vs I/R.

**50% SI**	**Sham**	**I/R**	**I/R+PSTI**
MPO (U/μg of protein)	0.161 ± 0.007	0.781 ± 0.009 **	0.266 ± 0.023 ** $ $
MDA (nmol/μg of protein)	0.238 ± 0.007	2.122 ± 0.08 **	0.895 ± 0.05 ** $ $
Hsp70 (pg/μg total protein)	18.88 ± 0.54	30.74 ± 0.4 **	37.33 ± 0.2 ** $
ICAM-1 (pg/μg total protein)	1.351 ± 0.306	2.154 ± 0.287 *	2.170 ± 0.355 *
**Lung**	**Sham**	**I/R**	**I/R+PSTI**
MPO (U/μg of protein)	0.171 ± 0.019	1.453 ± 0.05 **	0.7495 ± 0.028 ** $ $
MDA (nmol/μg of protein)	0.277 ± 0.027	1.073 ± 0.03 **	0.649 ± 0.034 ** $ $
Hsp70 (pg/μg total protein)	20.95 ± 1.10	30.48 ± 0.81 **	39.73 ± 0.88 ** $ $
ICAM-1 (pg/μg total protein)	3.52 ± 0.53	5.93 ± 0.16 **	5.67 ± 0.13 **
**KIDNEY**	**Sham**	**I/R**	**I/R+PSTI**
MPO (U/μg of protein)	0.157 ± 0.013	0.509 ± 0.048 **	0.291 ± 0.009 ** $ $
MDA (nmol/μg of protein)	0. 29 ± 0.023	0.55 ± 0.049 **	0.47 ± 0.047 **
Hsp70 (pg/μg total protein)	23.6 ± 1.02	34.1 ± 1.11 **	36.6 ± 2.96 **
ICAM-1 (pg/μg total protein)	5.1 ± 0.14	4.9 ± 0.16	5.22 ± 0.42
**LIVER**	**Sham**	**I/R**	**I/R+PSTI**
MPO (U/μg of protein)	1.99 ± 0.03	2.22 ± 0.05 **	2.06 ± 0.05 $ $
MDA (nmol/μg of protein)	0.24 ± 0.013	0.34 ± 0.009 **	0.27 ± 0.011 * $ $
Caspase 3 activity (Absorbance change)	0.023 ± 0.002	0.025 ± 0.002	0.021 ± 0.001
Caspase 9 activity (Absorbance change)	0.022 ± 0.001	0.023 ± 0.001	0.022 ± 0.001

Histological examination of renal and liver tissue (S2 Fig) showed minimal changes although biochemical assessment showed a small but significant increase in both MDA and MPO levels following I/R, with PSTI treatment significantly reducing MPO levels (Table 1).

#### Plasma human PSTI and cytokine levels

**Fig 4:**
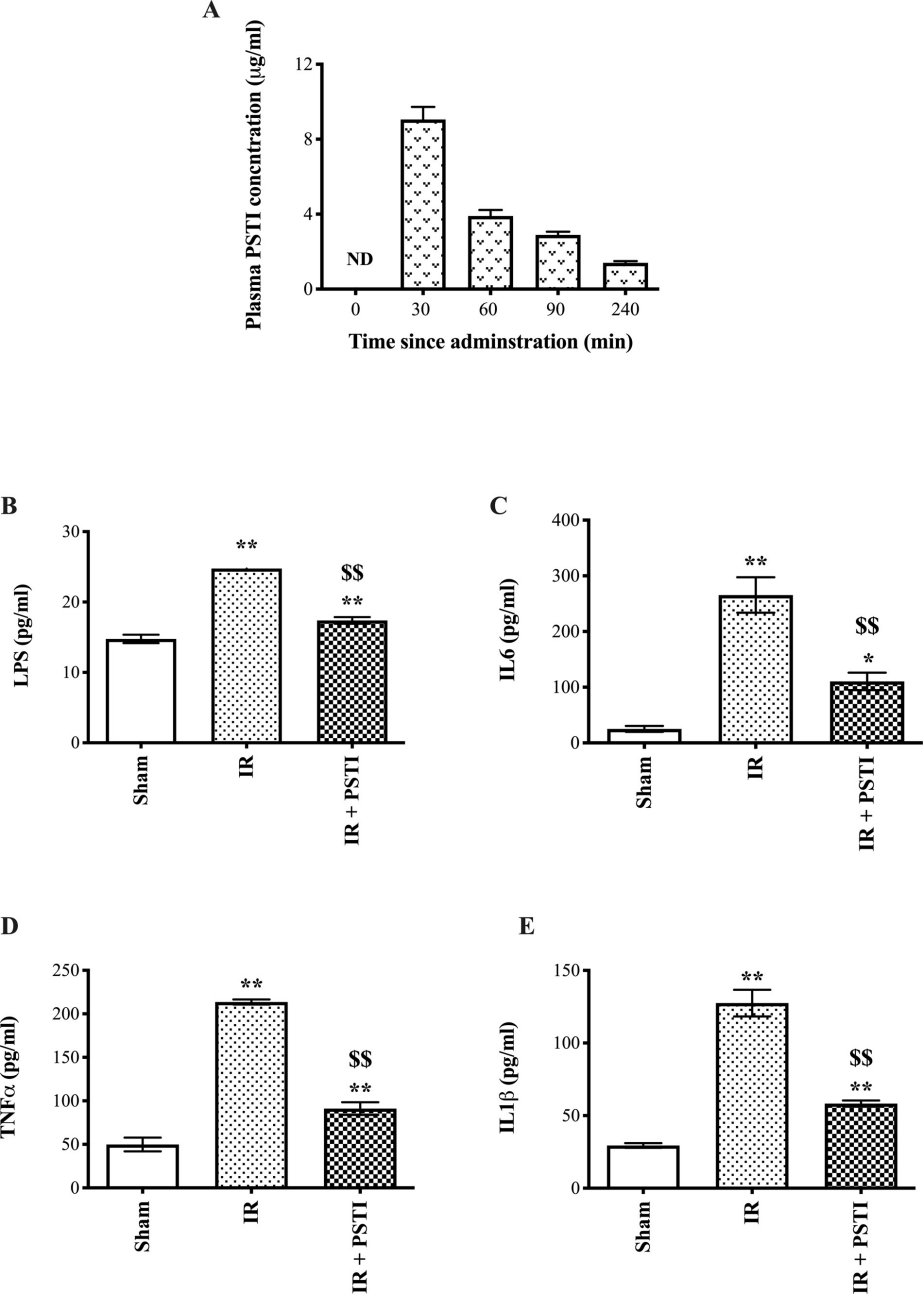
Plasma human PSTI, LPS and cytokine levels in animals undergoing I/R. **A.** Animals were injected with 20mg/kg human PSTI (ip). Blood was collected at various time points following injection for human PSTI analyses. ND = not detectable. B-E. shows changes in plasma LPS and cytokines at the end of the I/R or sham procedure protocol (equivalent to 240 min time point in A). Animals underwent I/R +/- PSTI given 60 min prior. At the end of the protocol, animals were killed, and blood collected for LPS (**B**) and cytokine analyses, IL6 (**C**), TNFα (**D**) and IL1β (**E**). * and ** signifies p<0.05 and p < 0.01 vs sham operated animals, $ $ signifies p < 0.01 vs I/R alone.

Plasma levels of human PSTI showed a peak of 9 μg/ml after 30 minutes with a progressive fall of to 1.4 μg/ml after 4 h (Fig 4A). Measurement of LPS and cytokines at the end of the I/R protocol showed that I/R caused significant increases in LPS, IL6, TNFα and IL1β which were all truncated in animals that had received PSTI pretreatment (Fig 4).

### Study series 4. Mechanisms of action of PSTI in the in vitro and in vivo studies

**Fig 5:**
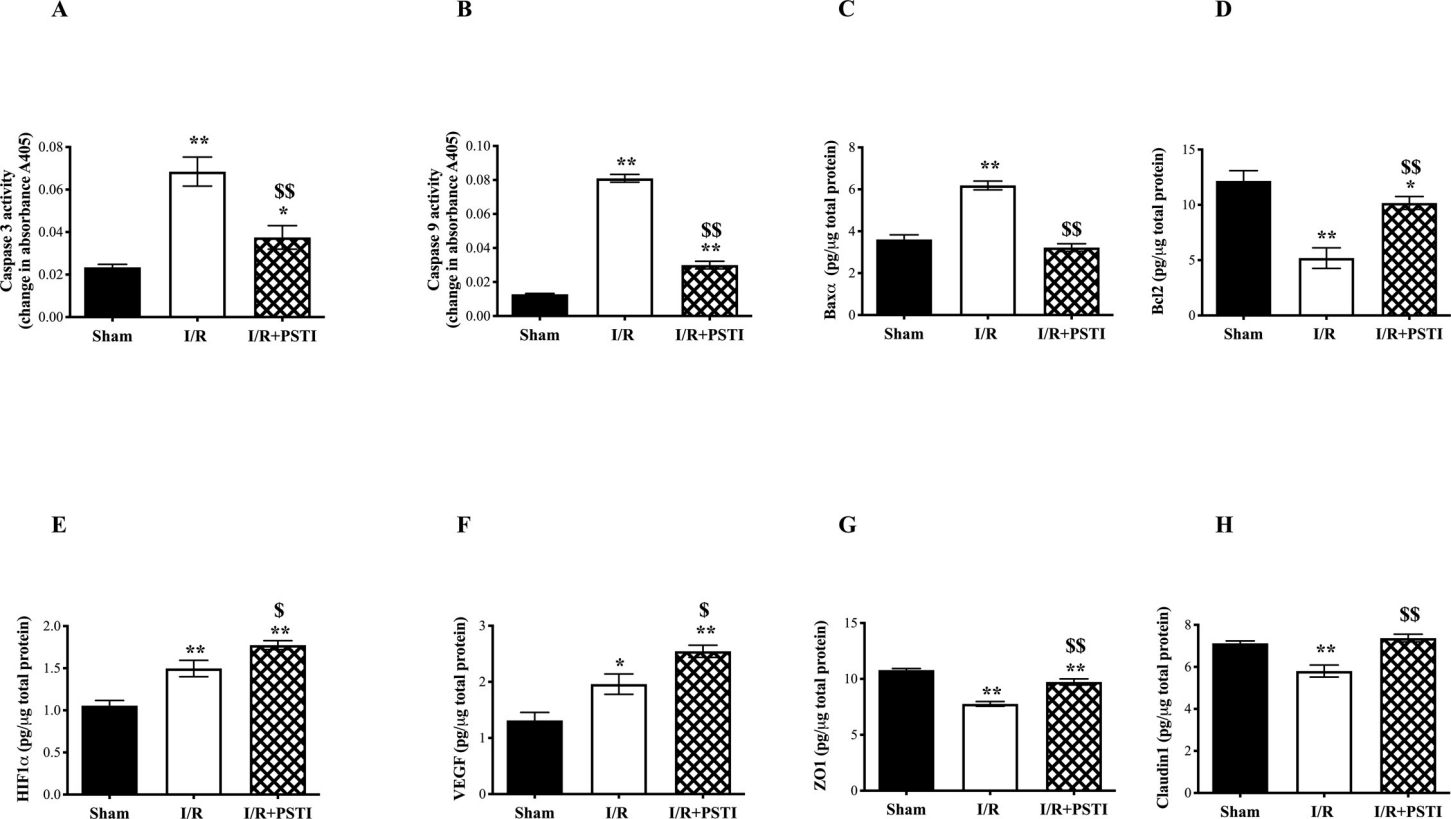
Molecular mechanisms of action of PSTI on mice SI undergoing I/R. Cleared cell lysates from the 50% SI site of animals that had undergone sham laparotomy or mesenteric I/R +/- PSTI pre-treatment (as in Fig 3), were analysed for changes in apoptotic molecules Caspase 3 (A), Caspase 9 (B), Baxα (C) and Bcl2 (D). HIF1α (E), VEGF (F), and the tight junction molecules ZO1 (G) and Claudin1 (H) levels were also analysed. * or ** signifies p <0.05 and < 0.01 vs sham operated animals, $ $ signifies p < 0.01 when comparing the effect of PSTI pre-treatment against animals that had undergone I/R without PSTI. No changes were seen measuring Caspase 3, 9, Bcl2, Bax, HIF1α, VEGF, ZO1 or Claudin 1 in the lungs or kidney of the same animals (not shown).

Results from N/H studies using cell culture and rat mesenteric I/R tissue gave similar results (Fig 5 and S1 Table) and are therefore discussed together.

Subjecting cells or 50% SI tissue to periods of hypoxia/ischemia followed by normoxia/reperfusion caused:

- Increased pro-apoptotic molecules Caspase 3 & 9
- Increased pro-apoptotic Baxα
- Reduced anti-apoptotic peptide Bcl2
- Reduced tight junction proteins ZO1 and Claudin 1

And

- Increased cell adhesion molecule ICAM-1
- Increased HIF1α & VEGF

I/R also caused increased Hsp70 in the lungs and kidneys and increased ICAM-1 in the lungs (Table 1). In the same animals, no changes were seen in Caspase 3, 9, Bcl2 or Baxα in the non-clamped region (90% SI) nor in lung, kidney or liver (Table 1).

PSTI pre-treatment of cells or animals caused:

- Truncation of rise pro-apoptotic molecules Caspase 3 & 9
- Truncation of rise of pro-apoptotic Baxα
- Truncation of fall in anti-apoptotic peptide Bcl2
- Truncation of fall in tight junction proteins ZO1 and Claudin 1
- Did not affect the increase in cell adhesion molecule ICAM-1
- Caused further increase in HIF1α& VEGF

Pre-treatment of animals with PSTI prior to I/R caused additional increases in Hsp70 in the lungs but did not affect I/R induced changes of ICAM-1 in the lungs (Table 1).

## Discussion

PSTI is a potent serine protease inhibitor initially identified in the pancreas where it helps prevent premature activation of pancreatic proteases [9]. Its wider distribution, including upregulation in damaged regions of the small intestine, suggests it also plays additional roles [10, 11]. Using a combination of in vitro and in vivo models we have now shown that PSTI reduced I/R gut injury and identified likely cellular pathways involved.

We previously showed that human PSTI stimulates migration of human colonic HT29 cells across wounded monolayers, reflecting the earliest stages of mucosal repair processes [12]. We now show that PSTI reduces hypoxia-normoxia-induced cell death in human stomach (AGS) and colonic (Caco-2) cells as well as the rat small intestinal cell line RIE1 in a dose and time dependant manner. These results demonstrate our findings have wide applicability across the gastrointestinal tract and are not species dependent, although caution always must be shown when extrapolating from in vitro cancer cell lines to the human in vivo situation.

Some of the earliest pathophysiological processes that occur due to I/R are mucosal epithelial cell damage, increased apoptosis, loss of basement membrane integrity and disruption of barrier function. Unless rapidly repaired, this promotes bacterial and luminal content translocation with induction of local production of cytokines [19]. To examine this early phase of I/R and MOF injury, we undertook a series of in vitro studies examining the effect of hypoxia-normoxia stress using two well-validated complementary models to examine changes in transepithelial resistance [18]. Although PSTI had no effect on TEER or HRP permeation when added to cells under normoxic conditions, it truncated the damaging effect of hypoxia-normoxia on epithelial integrity by about 65%, mirroring results seen following changes in cell survival.

Several different in vivo methods are available for inducing gut I/R (see ref [23]). These models fall into two general categories, the first involves complete temporary occlusion of the anterior mesenteric artery followed by a period of reperfusion [24], and the second involves segmental mesenteric occlusion by cross clamping the gut, causing obstruction of both arteries and veins. We chose this second method as it has the advantages of more closely reproducing the pathophysiology of strangulated bowel, greater reproducibility of injury [23], and easy identification of intestine outside of the I/R area that can be used as a control to differentiate local versus systemically mediated injury. As expected, the most severe damage was in the intestinal segment that had undergone direct I/R whereas the non-clamped terminal ileal region showed normal histology. Systemic injury was confirmed with lung tissue having marked acute inflammatory infiltrate with loss of lung architecture. Biochemical analyses confirmed histological findings with markedly raised MPO (neutrophil infiltration) and MDA (lipid peroxidation) levels in affected gut and lung tissue with a smaller but significant rise also seen in kidney tissue. Onset of lung injury is known to occur rapidly following I/R stress with liver and kidney injury manifesting later [25]. Our study protocol examined the early stages of I/R injury, probably explaining why lung damage predominated over liver and kidney injury. In addition, local activation of lung alveolar macrophages is an important damaging process in mediating lung injury [26]. Pre-treatment with PSTI significantly reduced histological and biochemical markers of injury in gut, lung and kidney.

We measured active caspase-3 (an effector caspase) and active caspase-9 (an initiator caspase) as markers of apoptosis and showed a 3-6-fold increase in apoptosis in the in vivo model, with this rise being virtually eliminated if PSTI was pre-administered. PSTI truncated the increase in the pro-apoptotic signalling molecule Baxα and maintained levels of the anti-apoptotic molecule Bcl-2 in the damaged segments, probably contributing to this anti-apoptotic effect. These effects were mediated locally as distant organs and non-clamped regions of bowel did not show any change in caspase, Bcl-2 or Baxα activity. Hsp70, a protective protein against apoptosis [27], increased in response to PSTI when given to cells or in the gut, lung and kidneys of animals given PSTI under normoxic conditions, further rise seen when exposed to I/R. HIF1α and VEGF expression reduces apoptosis, stimulates angiogenesis and causes immune modulation [28, 29]. HIF1α and VEGF increased in response to PSTI in the gut region that had undergone I/R but not in distant sites. The current studies therefore suggest that Hsp70, HIF1α and VEGF may all have relevance to the protective effects of PSTI.

ICAM-1 stabilises cell-cell interactions and facilitates leukocyte endothelial transmigration. Its upregulation in the lungs resulting from gut I/R is thought be an important signalling mechanism involved in pulmonary leukocyte infiltration [30]. PSTI reduced neutrophil infiltration of the lungs but did not change the rise in ICAM-1 caused by I/R, suggesting that the pulmonary protective effects of PSTI must be through other processes.

Intestinal epithelial tight junctions are multiprotein complexes that act as selective barriers. The fall in ZO1 and Claudin1 seen in the clamped I/R region but not distant organs, results in lowered intestinal integrity and increased permeability of the affected gut. The truncation of these changes by PSTI can be considered potentially beneficial and may have contributed to the enhanced integrity seen in the TEER and HRP permeability studies.

The identity of the PSTI receptor(s) involved in these effects is unclear, Niinobu [31] reported ^125^I-labeled PSTI-binding sites on a variety of cell lines, including human skin fibroblasts (BUD-8) and colon cancer (HCT-15) cells. These binding sites were able to be saturated and displaced by excess noniodinated PSTI but not by EGF [31], were not cell surface proteinases, and the receptor ligand complex had a molecular weight of ∼140 kDa. Our previous studies have shown a close relationship between PSTI and the EGF-receptor; the pro-migratory effects of PSTI is inhibited by an EGFR-neutralizing antibody [13], deletion of EGFR prevents the promigratory activity of PSTI in human colon cancer cells and phosphorylation of EGFR occurs in response to PSTI [12]. Taken together, these results suggest that the relationship between PSTI and EGFR is probably not that of a direct receptor ligand but is inducing cross-phosphorylation of EGFR and/or influencing its downstream pathways.

Binding of PSTI to its putative receptor causes phosphorylation of ERK and JNK1, whose functions include influencing apoptosis and the inflammatory response, and RSK1, which is involved in transcriptional regulation by phosphorylating c-Fos and CREB [32]. ERK1 and ERK2 (also known as MAPK3 and MAPK1) are part of the Ras-Raf-ERK signal transduction cascade often found downstream of growth factor receptor activation. Phosphorylation of ERK1 and ERK2 causes multiple effects on transcription factors and scaffolding proteins [33, 34]. PSTI also stimulates phosphorylation of Akt2 which is involved in cell migration and invasion [35] but does not phosphorylate Akt1, probably explaining the lack of proliferative activity [13].

Plasma levels of human PSTI reached a peak of 9 μg/ml in the current studies. These were approximately 1,000 times higher than normal circulating PSTI levels in healthy humans [36], although PSTI acts as an acute phase reactant reaching levels roughly three-fold higher than in healthy subjects [37]. The results from our studies are therefore of pharmacological rather than physiological relevance. There is always concern about the use of growth factor therapy in stimulating cancer development in distant organs, especially if given systemically. PSTI has the major potential advantage of stimulating repair without stimulated proliferation when tested against a wide range of cancer cell lines by ours and other groups [12].

In summary, our studies showed that PSTI protects gut and distant organs from mesenteric I/R-induced injury. Mechanisms involved included reduction in pro-apoptosis signalling, increased Hsp70 and VEGF production, and maintaining levels of tight junction proteins. PSTI may therefore provide a novel approach to the prevention and treatment of a wide variety of gut conditions which have I/R as a component of their pathophysiology. Further studies including clinical trials appear warranted.

## Supporting information

S1 Fig Effect of different times of hypoxia (ischemia) and return to normoxia (reperfusion) on cell viability. Cell were exposed to hypoxia for 1h (O), 4h (□) or 8h (Δ), and returned to normoxia (reperfusion) for 1,4,8 or 24 h in the presence (black symbols) and absence of glucose (open symbols) in the medium during the hypoxic period. Cell viability (A, MTT assay) and damage (B, LDH activity) were then determined. Results shown are for AGS cells, RIE1 and Caco-2 cells gave similar results. Cells exposed to hypoxia in the presence of glucose showed no significant change in survival or damage compared to normoxic controls (black symbols). In contrast, cells incubated in medium without glucose showed significant damage and decreased viability (open symbols).
(TIFF)

S2 Fig Effect of PSTI on kidney and Liver injury. Mice underwent a sham (laparotomy only) procedure or subjected to 30 min mesenteric ischemia followed by 3 hours of reperfusion (I/R). Some animals also received PSTI (20 mg/kg, ip) 1 hour before gut clamping (I/R + PSTI). Photomicrographs (original magnification 200X, scale bar = 100 μM) of liver tissue from **A)** sham operated control animal, **B)** animal that had undergone I/R protocol, **C)** animal pre-treated with PSTI prior to I/R. Photomicrographs of kidney tissue (original magnification 200X, scale bar = 100 μM) from **D)** sham operated control animal, **E)** animal that had undergone I/R protocol, **F)** animal pre-treated with PSTI prior to I/R.
(TIFF)

S1 Table Effect of hypoxia-normoxia—+/- pre-administration of PSTI on injury & apoptotic and protective pathways in Caco2 cells. Cells were exposed to 4 h hypoxia followed by 24 normoxia. Data presented as mean +/- SEM. ** signifies p <0.01 vs normoxia alone, $ and $ $ signifies p<0.05 and <0.01 vs N/H alone. Addition of PSTI to cells under normoxic conditions throughout had no effect on any of the pathways except for Hsp70 (see Table). Cells exposed for 1 h hypoxia and 24 normoxia showed similar changes to those shown. Similar results were also seen using AGS and RIE cell lines.
(DOCX)

## Abbreviations

Baxα: B cell leukemia/lymphoma-2 associated X protein-alpha
Bcl-2: B-cell lymphoma 2
HRP: horse radish peroxidase
H/N: hypoxia/normoxia
I/R: ischeamia/reperfusion
LDH: lactate dehydrogenase
MDA: malondialdehyde
MOF: multi organ failure
MPO: myeloperoxidase
MTT: 3-(4,5-: di: methylthiazol-2-yl)-2,5-diphenyltetrazolium bromide
PSTI: pancreatic secretory trypsin inhibitor
TEER: transepithelial resistance
TJ: tight junction
ZO1: zona occludens protein
