# Supplementary figures and images for: Pancreatic secretory trypsin inhibitor reduces multi-organ injury caused by gut ischemia/reperfusion in mice
Source: PLoS One. 2020 Jan 10;15(1):e0227059. doi: 10.1371/journal.pone.0227059 (PMC6953855; doi:10.1371/journal.pone.0227059)

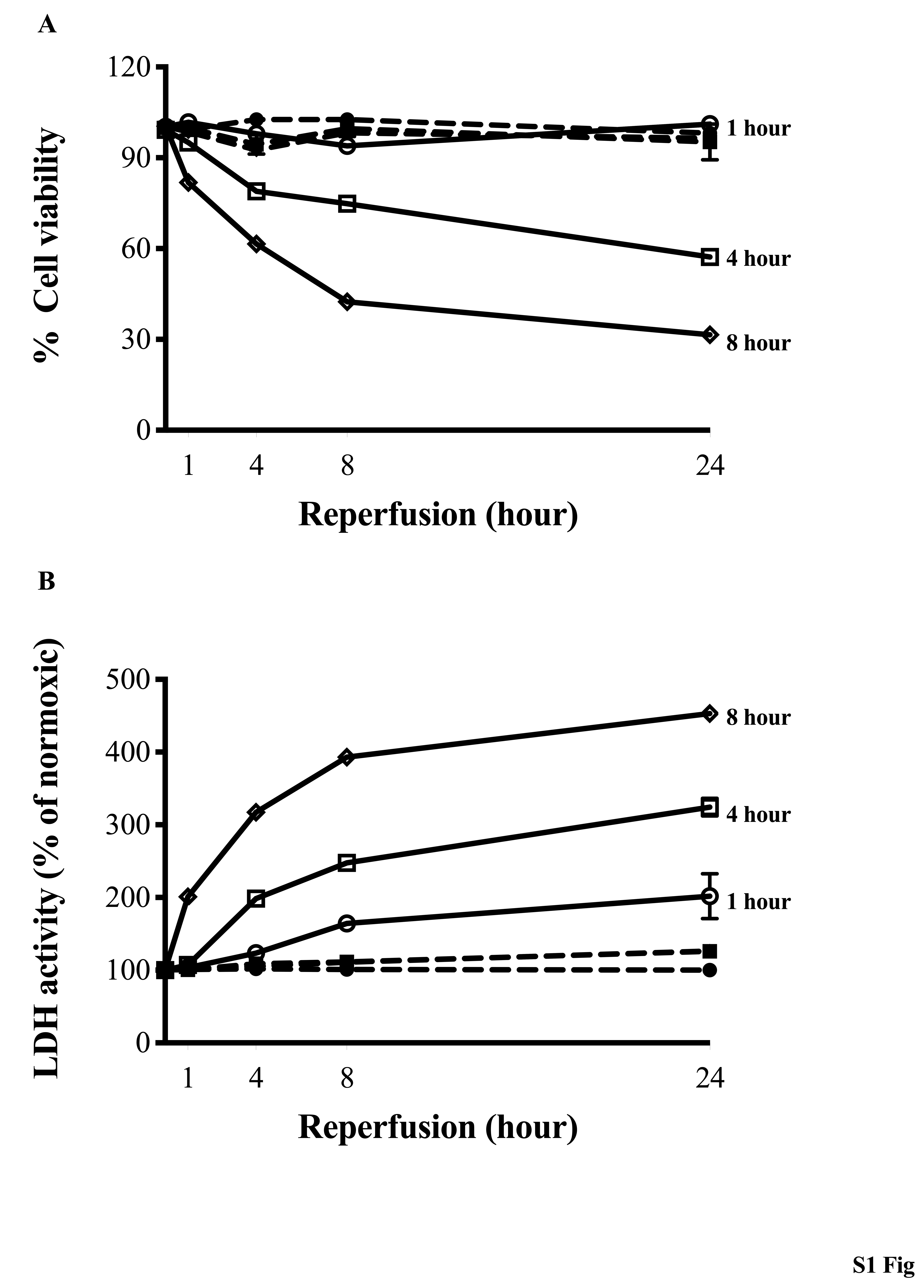

Supplement: S1 Fig — Cell were exposed to hypoxia for 1h (O), 4h (□) or 8h (Δ), and returned to normoxia (reperfusion) for 1,4,8 or 24 h in the presence (black symbols) and absence of glucose (open symbols) in the medium during the hypoxic period. Cell viability (A, MTT assay) and damage (B, LDH activity) were then determined. Results shown are for AGS cells, RIE1 and Caco-2 cells gave similar results. Cells exposed to hypoxia in the presence of glucose showed no significant change in survival or damage compared to normoxic controls (black symbols). In contrast, cells incubated in medium without glucose showed significant damage and decreased viability (open symbols). (TIFF) [file pone.0227059.s001.tiff]

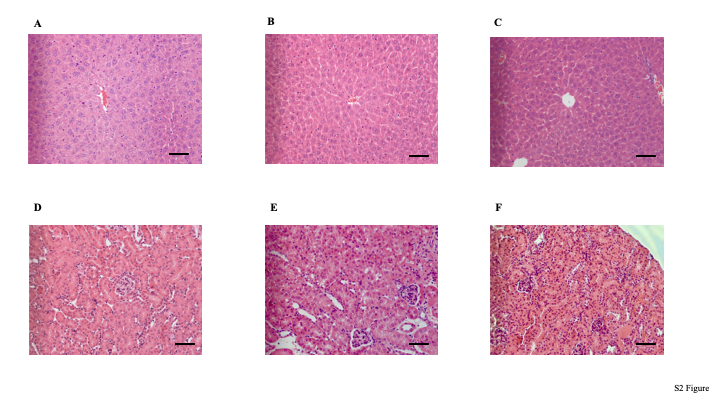

Supplement: S2 Fig — Mice underwent a sham (laparotomy only) procedure or subjected to 30 min mesenteric ischemia followed by 3 hours of reperfusion (I/R). Some animals also received PSTI (20 mg/kg, ip) 1 hour before gut clamping (I/R + PSTI). Photomicrographs (original magnification 200X, scale bar = 100 μM) of liver tissue from A) sham operated control animal, B) animal that had undergone I/R protocol, C) animal pre-treated with PSTI prior to I/R. Photomicrographs of kidney tissue (original magnification 200X, scale bar = 100 μM) from D) sham operated control animal, E) animal that had undergone I/R protocol, F) animal pre-treated with PSTI prior to I/R. (TIFF) [file pone.0227059.s002.tiff]
