# Supplementary material for: Pancreatic secretory trypsin inhibitor reduces multi-organ injury caused by gut ischemia/reperfusion in mice
Source: PLoS One. 2020 Jan 10;15(1):e0227059. doi: 10.1371/journal.pone.0227059 (PMC6953855; doi:10.1371/journal.pone.0227059)
Supplement: S1 Table — Cells were exposed to 4 h hypoxia followed by 24 normoxia. Data presented as mean +/- SEM. ** signifies p <0.01 vs normoxia alone, $ and $ $ signifies p<0.05 and <0.01 vs N/H alone. Addition of PSTI to cells under normoxic conditions throughout had no effect on any of the pathways except for Hsp70 (see Table). Cells exposed for 1 h hypoxia and 24 normoxia showed similar changes to those shown. Similar results were also seen using AGS and RIE cell lines. (DOCX) [file pone.0227059.s003.docx]

| **Caco2** | **Normoxia**  **throughout** | **N/H alone** | **N/H+PSTI** |
| --- | --- | --- | --- |
| Caspase 3 activity  (Change in absorbance) | 0.063 + 0.004 | 0.0995 + 0.004  ** | 0.075 + 0.002  ** $ |
| Caspase 9 activity  (Change in absorbance) | 0.066 + 0.002 | 0.0975 + 0.005  ** | 0.0795 + 0.002  ** $$ |
| Bcl2 (pg/µg total protein) | 617.5 + 2.89 | 327.5 + 10.1  ** | 515.0 + 17.32  ** $$ |
| Baxα(pg/µg total protein) | 141.8 + 0.54 | 286.5 + 0.09  ** | 251.5 + 2.7  ** $$ |
| HIF1α (pg/µg total protein) | 76.76 + 1.31 | 95.4 + 0.01  ** | 102.7 + 0.525  ** $$ |
| VEGF (pg/µg total protein) | 349.97 + 11.54 | 472.48 + 18.76  ** | 532.47 + 1.44  ** $ |
| Hsp70 (pg/µg total protein) | 455 + 2.887  **Normoxia+PSTI**  640 + 5.774  ** | 745 + 2.887  ** | 805 + 2.88  ** $$ |
| ICAM-1 (pg/µg total protein) | 440.6 + 1.237 | 552.1 + 0.412  ** | 547.8 + 1.237  ** |
| ZO1 (pg/µg total protein) | 8.1 + 0.036 | 4.44 + 0.115  ** | 6.46 + 0.011  ** $$ |
| Claudin 1 (pg/µg total protein) | 7.69 + 0.029 | 3.91 + 0.121  ** | 6.26 + 0.069  ** $$ |

**S1 Table - Effect of hypoxia-normoxia - +/- pre-administration of PSTI on injury & apoptotic and protective pathways in Caco2 cells.** Cells were exposed to 4 h hypoxia followed by 24 normoxia. Data presented as mean +/- SEM. ** signifies p <0.01 vs normoxia alone, $ and $$ signifies p<0.05 and <0.01 vs N/H alone. Addition of PSTI to cells under normoxic conditions throughout had no effect on any of the pathways except for Hsp70 (see Table). Cells exposed for 1 h hypoxia and 24 normoxia showed similar changes to those shown. Similar results were also seen using AGS and RIE cell lines.
